# Thoracic Outlet Syndrome: Fingertip Cannot Replace Forearm Photoplethysmography in the Evaluation of Positional Venous Outflow Impairments

**DOI:** 10.3389/fphys.2021.765174

**Published:** 2021-11-23

**Authors:** Jeanne Hersant, Pierre Ramondou, Sylvain Durand, Mathieu Feuilloy, Mickael Daligault, Pierre Abraham, Samir Henni

**Affiliations:** ^1^Vascular Medicine, University Hospital, Angers, France; ^2^UMR CNRS 1083 INSERM 6214, LUNAM University, Angers, France; ^3^Sports and Exercise Medicine, University Hospital, Angers, France; ^4^EA 4334 Motricité Interaction Performance, Le Mans University, Le Mans, France; ^5^UMR CNRS 6613 LAUM, Le Mans, France; ^6^School of Electronics (ESEO), Angers, France; ^7^Department of Thoracic and Vascular Surgery, University Hospital, Angers, France

**Keywords:** thoracic outlet syndrome (TOS), photoplethysmography (PPG), forearm, veins, fingertip, pathophysiology, movement (MeSH)

## Abstract

**Objective:** Fingertip photoplethysmography (PPG) resulting from high-pass filtered raw PPG signal is often used to record arterial pulse changes in patients with suspected thoracic outlet syndrome (TOS). Results from venous (low-pass filtered raw signal) forearm PPG (V-PPG) during the Candlestick-Prayer (Ca + Pra) maneuver were recently classified into four different patterns in patients with suspected TOS, two of which are suggestive of the presence of outflow impairment. We aimed to test the effect of probe position (fingertip vs. forearm) and of red (*R*) vs. infrared (*IR*) light wavelength on V-PPG classification and compared pattern classifications with the results of ultrasound (US).

**Methods:** In patients with suspected TOS, we routinely performed US imaging (US + being the presence of a positional compression) and Ca + Pra tests with forearm V-PPG_*IR*_. We recruited patients for a Ca + Pra maneuver with the simultaneous fingertip and forearm V-PPG_*R*_. The correlation of each V-PPG recording to each of the published pattern profiles was calculated. Each record was classified according to the patterns for which the coefficient of correlation was the highest. Cohen’s kappa test was used to determine the reliability of classification among forearm V-PPG_*IR*_, fingertip V-PPG_*R*_, and forearm V-PPG_*R*_.

**Results:** We obtained 40 measurements from 20 patients (40.2 ± 11.3 years old, 11 males). We found 13 limbs with US + results, while V-PPG suggested the presence of venous outflow impairment in 27 and 20 limbs with forearm V-PPG_*IR*_ and forearm V-PPG_*R*_, respectively. Fingertip V-PPG_*R*_ provided no patterns suggesting outflow impairment.

**Conclusion:** We found more V-PPG patterns suggesting venous outflow impairment than US + results. Probe position is essential if aiming to perform upper-limb V-PPG during the Ca + Pra maneuver in patients with suspected TOS. V-PPG during the Ca + Pra maneuver is of low cost and easy and provides reliable, recordable, and objective evidence of forearm swelling. It should be performed on the forearm (close to the elbow) with either PPG_*R*_ or PPG_*IR*_ but not at the fingertip level.

## Introduction

Avenous origin is proposed as the second most frequent etiology of thoracic outlet syndrome (V-TOS) (Illig et al., 2016). Beyond effort venous thrombosis (Moore and Wei Lum, 2015; Cook and Thompson, 2021), transient positional compression of the subclavicular vein may result in venous outflow impairment during arm elevation, leading to positional upper limb pain and/or swelling (i.e., McCleery syndrome). Despite the absence of thrombosis, there is evidence that in McCleery syndrome, symptoms resulting from these positional outflow impairments can be improved with appropriate treatments (Likes et al., 2014; Moore and Wei Lum, 2015; Ryan et al., 2018; Wooster et al., 2019). Ultrasound (US) investigation is difficult in V-TOS, even in cases of chronic occlusion (Paget Schroetter syndrome) and does not measure positional upper limb volume changes (Jourdain et al., 2016; Brownie et al., 2020).

Photoplethysmography (PPG) is a low-cost and fairly established technique that estimates volume changes from the absorbance of red (*R*) or infrared (*IR*) light by illuminated tissues. There are two components within the raw PPG signal (Hickey et al., 2015). A high-pass filter can evaluate the small volume changes due to arterial pulsatility (A-PPG), while a low-pass filter removes the arterial pulsatility to assess limb volume changes mainly resulting from venous volume changes (V-PPG). It must be kept in mind that PPG is a semiquantitative technique with absolute values or absolute changes, being highly variable in test–retest recordings. We recently reported our experience with low-pass filtered reflectance infrared light forearm PPG (V-PPG_*IR*_) during the Candlestick-Prayer (Ca + Pra) maneuver (Hersant et al., 2021). Our specific interest in the prayer position is because it can completely empty elevated upper limbs by opening the costoclavicular angle and thus might confirm whether venous outflow was impaired during the candlestick position. Therefore, the V-PPG signal varies between complete filling (arm lowered used as zero value) to complete emptying (upper limb elevated in the prayer position, used as 100% value). This approach seeks to normalize results and thereby improve the interpretation of this otherwise semiquantitative technique.

Fingertip A-PPG_*R*_ resulting from high-pass filtered raw PPG_*R*_ signal is often used to record arterial pulse changes in patients with suspected TOS (Colon and Westdorp, 1988; Baxter et al., 1990; Kleinert and Gupta, 1993). Using a low-pass filter to remove the arterial pulsatility, the resulting fingertip V-PPG_*R*_ might appear as an attractive tool for confirming the presence of upper-limb swelling (venous volume increase) during positional maneuvers in TOS. Since the V-PPG_*IR*_ probes were positioned on the forearm close to the elbow in our initial experiment, we aimed to test the hypothesis that patterns observed at the forearm level could be found at the fingertip level. Then, we recorded fingertip and forearm V-PPG_*R*_ simultaneously. Since light wavelength is described as an important determinant of the PPG responses (Chatterjee et al., 2018), we also aimed to compare forearm V-PPG_*R*_ results to those obtained through forearm V-PPG_*IR*_.

In brief, this study was performed to test the influence of probe position (fingertip vs. forearm) and light wavelength (red vs. infrared) over V-PPG patterns and aimed to compare V-PPG results to the results of US imaging.

## Materials and Methods

### Population

A prospective study was performed among patients who were referred to our laboratory from January 1 to December 31, 2020 for the investigation of symptoms suggesting the presence of TOS with upper-limb US imaging. As a routine during each visit, we recorded patient demographics and conditions, including age, sex, weight, height, history of the chest, shoulder or arm trauma or surgery, professional activity, and any ongoing treatments. All patients had bilateral forearm V-PPG_*IR*_ recording (2–3 cm distal to the elbow crease) with Vasolab320 (ELCAT^®^, Germany), as previously described (Hersant et al., 2021). In brief, it is stated that the Ca + Pra maneuver was performed with four consecutive phases: arm elevation to the candlestick position (in <5 s), maintenance of the “candlestick” (Ca) position until 30 s, rapid change to the “prayer” (Pra) position, which is maintained for 15 s, and then arm lowering (Hersant et al., 2021). For routine PPG_*IR*_, after one training session, we waited for at least 1 min at rest with the arms along the torso before commencing recording (Hersant et al., 2021). It should be noted that ELCAT^®^ starts from zero and records V-PPG_*IR*_ values in arbitrary units (AUs) at a sample rate of 4 Hz for 60 s and then automatically stops. It is also worth noting that increases in values correspond to decreases in volume. In parallel, US investigations were performed by trained operators, and maneuvers were left to the choice of the operator. US results were encoded limb by limb and considered positive (US^+^) when the report explicitly described positional compression (or occlusion) of the subclavicular vein regardless of which maneuver was performed. Otherwise, the US result was recorded as negative (US^–^) for venous compression. US and PPG_*IR*_ were systematically performed by different operators blinded to the results of the other test. All demography and clinical results were registered in an ethically approved database. Patients denying the use of their data, unable to understand the information for linguistic or cognitive reasons, and patients under 18 years of age were not recorded in the database and were considered to be ineligible for inclusion in the STOUT study.

### Methods

**FIGURE 1:**
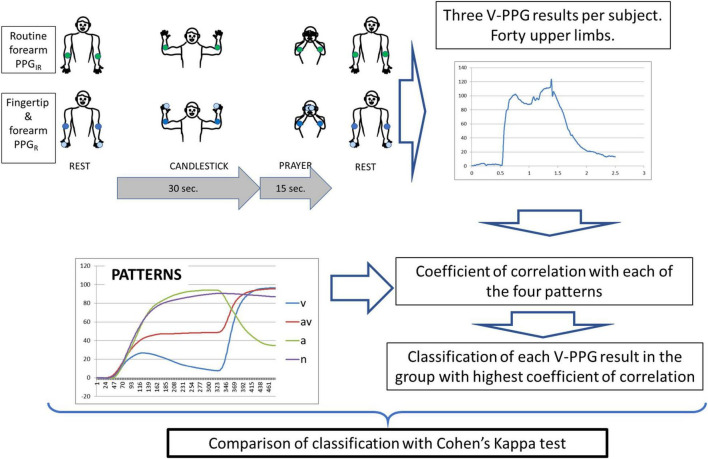
Schematic representation of the methods.

The STOUT study was settled to allow the development of a homemade specific device allowing the recording of V-PPG_*R*_ simultaneously at the forearm and fingertip levels and on both sides. The protocol was promoted by the university hospital in Angers, approved by the ethics committee, and registered in the clinicaltrial.gov website under identifier NCT03355274, before first inclusion. Patients were eligible to participate in this study if they fulfilled the inclusion and exclusion criteria (Table 1). Patients that agreed to participate provided signed informed consent after oral and written explanation of the protocol. The protocol and all related procedures were performed in compliance with the principles outlined in the Declaration of Helsinki. It is noted that inclusions were suspended during the coronavirus disease (COVID) wave. A schematic representation of the methods of this study is presented in Figure 1.

**TABLE 1:** Eligibility criteria for this study.

Inclusion Criteria:	• Age >18 years old • Patients referred for investigation of thoracic outlet syndrome • Affiliation to the French National healthcare system • French-speaking patients • Ability to stand still for half a minute

Exclusion Criteria:	○ Pregnancy ○ Inability to understand the study goal ○ Patients protected by decision of law

**FIGURE 2:**
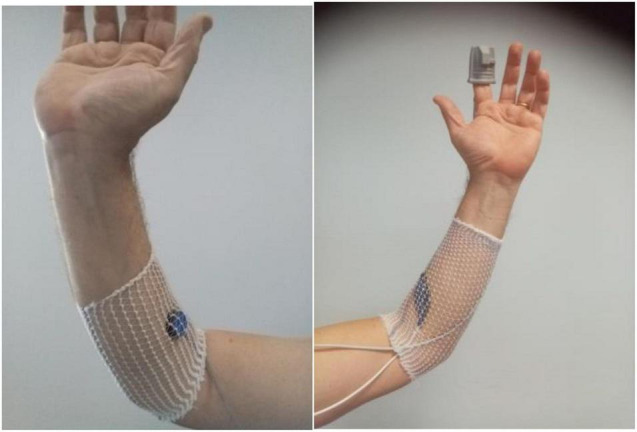
Illustration of the location of probes. The **left panel** shows the IR light V-PPG probe of the ELCAT device on the forearm close to the elbow crease; the **right panel** shows the probes at the fingertip and forearm level used during the red light V-PPG recordings.

In patients included in the STOUT protocol, red light V-PPG (V-PPG_*R*_) signals were recorded on both forearms and at the fingertip of the second finger of both hands, with the patient seated. Fingertip V-PPG_*R*_ was recorded with adult finger soft-tip SpO2 sensors (Sino-K, China) as shown in Figure 2. On the forearms, we used flat red-light forehead SpO2 sensors (Nellcor Mansfield, MA, United States) placed 2–3 cm distal to the elbow crease covered with a Surgifix net (Urgo, France).

The system recorded fingertip and forearm V-PPG_*R*_ values through a National instrument 4 channels 16-bit 9215 analog to digital converter, on both sides simultaneously at a sample rate of 200 Hz with a low pass filter of 0.2 Hz. The recording was started at least 15 s before the beginning of the Ca + Pra maneuver and stopped at least 1 min after the end of the procedure. Values are recorded in volts. Then, data were resampled to 4 Hz to allow comparison with ELCAT results.

For all PPG recordings, the Ca + Pra procedures were performed as previously reported (Figure 1) with the following four consecutive phases: arm elevation to the candlestick position (in <5 s), maintenance of the “candlestick” (Ca) position until 30 s, rapid change to the “prayer” (Pra) position that is maintained for 15 s, and then arm lowering.

### Statistical Analysis

All V-PPG results were expressed in normalized emptying units (NEUs) using the starting voltage as a zero (0 NEU: arm fully filled) and the highest voltage observed during the candlestick or prayer maneuver as full emptying (100 NEU). Since the method and speed of arm lowering with the ELCAT was not normalized and as the recording period was too short to allow the return toward baseline value for routine IR V-PPG_*IR*_, the analysis was performed over the first 45 s of the recording.

The data are presented as numbers (percentages), medians (25 and 75 percentiles), or means ± SD. Comparison of absolute values at the candlestick position and the prayer position with forearm V-PPG_*IR*_ and with fingertip and forearm V-PPG_*R*_ recordings were performed on a limb by limb basis, and the differences were tested with ANOVA and Scheffe post-tests.

We calculated the coefficient of correlation of each recording to the reference curve of each of the four previously published representative patterns (Hersant et al., 2021). The reference curve was the mean of all recordings included in the cluster. Then, each recording was classified in the pattern that showed the highest correlation coefficient. Following this, Cohen’s kappa test was used to analyze the concordance of classification observed between the forearm V-PPG_*IR*_, the fingertip V-PPG_*R*_, and the forearm V-PPG_*R*_. Kappa is always ≤1. It is generally agreed that kappa <0.00, 0.00–0.20, 0.21–0.40, 0.41–0.60, 0.61–0.80, and 0.81–1 indicate no, slight, fair, moderate, substantial, and almost perfect to perfect agreements, respectively (Landis and Koch, 1977). Kappa values are reported with SE (± SE) and the 95% CI (minimum to maximum values). All statistical analyses were performed using SPSS^®^ (IBM SPSS statistics version 15.0, Chicago, IL, United States) and Graphpad^®^ for online Kappa calculation (Graphpad software, San Diego, CA, United States)^1^. For all tests, a two-tailed *P* < 0.05 was considered to be statistically significant.

## Results

Among the included patients (*n* = 21), one patient did not have forearm V-PPG_*IR*_ recordings for technical reasons and was excluded from this study. The remaining 20 patients (40 upper limbs) were 40.2 ± 11.3 years old, 11 males and 9 females, 171 ± 8 cm in height, and 77.2 ± 12.5 kg in weight. Notably, 10 patients had a history of chest shoulder or arm surgery or trauma. Half of the patients were unemployed, 8 of them due to their upper limb pain. Nine patients took pain killers, of whom three patients did so, on a daily basis. Of note, 2 patients reported unilateral right pain, 7 reported unilateral left pain, and 11 reported bilateral pain. US imaging in medical files reported the presence of right unilateral (*n* = 2), left unilateral (*n* = 3), or bilateral (*n* = 4) venous positional compression, resulting in 14 US^+^ and 26 US^–^ limbs.

**FIGURE 3:**
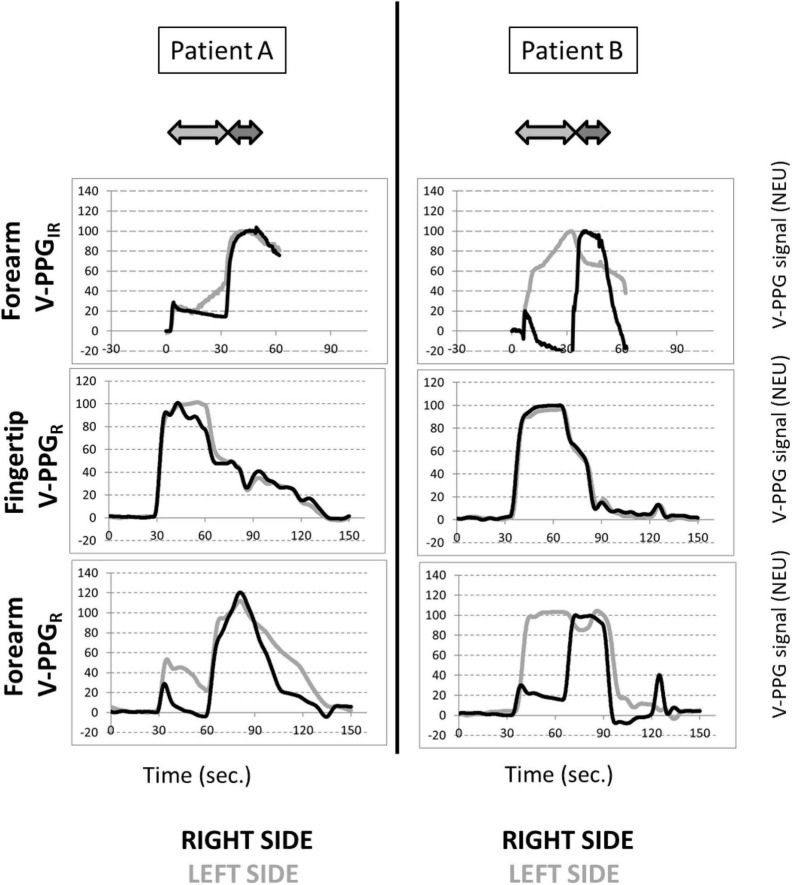
Example of recordings in a patient with bilateral symptoms (patient A) and unilateral left symptoms (patient B) during the candlestick (light gray arrow) and prayer (dark gray arrow) maneuvers. Zero is resting state with filled veins, and 100% is the maximal volume observed until the end of the prayer maneuver. Since a small overshoot was sometimes observed when moving the arms down after the prayer maneuver, some values may be in excess of 100%. It is noted that the system used for VPP_*IR*_ does not allow recordings in excess of 60 s.

Typical examples of recordings obtained for forearm V-PPG_*IR*_ and for the fingertip and forearm V-PPG_*R*_ test are presented in Figure 3. Of interest is that the outflow impairment found in both upper limbs of patient A and the left upper limb of patient B was observed with the forearm V-PPG_*R*_ and the forearm V-PPG_*IR*_ but was not visible at the hand level with fingertip V-PPG_*R*_.

On average, in the candlestick position, a significant difference was found between results at the fingertip level (88 ± 13 NEU) and those found during the forearm V-PPG_*IR*_ (56 ± 45 NEU), between the forearm V-PPG_*R*_ (53 ± 43 NEU) with *P* < 0.05, and between fingertip V-PPG_*R*_ and forearm results tests. This difference was not observed for the prayer position with values being 71 ± 16 NEU, 86 ± 38 NEU, and 94 ± 11 NEU, respectively.

It should be noted that none of the 40 fingertip V-PPG_*R*_ recordings was suggestive of the presence of outflow impairment (V or AV patterns). Table 2 reports the comparison of V-PPG classification for results obtained for the different recordings. As shown, forearm V-PPG_*IR*_ vs. forearm V-PPG_*R*_ showed fair agreement as follows: number of observed agreements: 18 (45.0% of the observations), kappa = 0.235 ± 0.103, and 95% CI [0.034–0.437]. Forearm V-PPG_*IR*_ vs. fingertip V-PPG_*R*_ showed no agreement as follows: number of observed agreements: 7 (17.5% of the observations), kappa = −0.020 ± 0.061, and 95% CI [−0.140–0.100]. Finally, forearm V-PPG_*R*_ vs. fingertip V-PPG_*R*_ showed slight agreement as follows: number of observed agreements: 13 (35.1% of the observations), kappa = 0.092 ± 0.053, and 95% CI [−0.012–0.196].

**TABLE 2:** Classification of the patterns observed with red (R) or IR venous photoplethysmography (V-PPG) for each studied limb (*n* = 40).

A	Forearm V-PPG_*IR*_					
	
		V	AV	A	N	*Total*
Forearm V-PPG_*R*_	V	9	2	2	3	**16**
	AV	1	2	1	0	**4**
	A	1	0	2	0	**3**
	N	5	5	2	5	**17**
	**total**	**16**	**9**	**7**	**8**	**40**

**B**	**Forearm V-PPG_*IR*_**					
	
		**V**	**AV**	**A**	**N**	***Total***

Fingertip V-PPG_*R*_	V	0	0	0	0	*0*
	AV	0	0	0	0	*0*
	A	5	2	3	4	*14*
	N	11	7	4	4	**26**
	**total**	**16**	**9**	**7**	**8**	**40**

**C**	**Forearm V-PPG_*R*_**					
	
		**V**	**AV**	**A**	**N**	***Total***

Fingertip V-PPG_*R*_	V	0	0	0	0	*0*
	AV	0	0	0	0	*0*
	A	8	2	0	4	*14*
	N	8	2	0	13	**26**
	**total**	**16**	**4**	**3**	**17**	**40**

*Tables A–C are forearm V-PPG_IR_ vs. forearm V-PPG_R_, forearm V-PPG_IR_ vs. fingertip V-PPG_R_, forearm V-PPG_R_ vs. fingertip V-PPG_R_. V, AV, A, and N groups that are assumed indicative of isolated outflow impairment, arterial inflow/venous outflow simultaneous impairment, isolated inflow impairment, and normal responses, respectively (see text for details).*

When compared with US, among the limbs with US^+^ (*n* = 13), nine upper limbs with forearm V-PPG_*IR*_ and eight limbs with forearm V-PPG_*R*_ had V-PPG patterns of V or AV type, but an additional 16 V or AV patterns were observed among forearm V-PPG_*IR*_, 12 V or AV patterns among of the forearm V-PPG_*R*_ recordings, and in the 27 US^–^ limbs. Globally, there were many more forearm V-PPG tests showing a venous outflow impairment than during US imaging. Again, it is important to note that none of the 40 fingertip V-PPG_*R*_ recordings was suggestive of the presence of venous outflow impairment.

## Discussion

The major results and points of interest of this study are as follows. First, we observed more V-PPG patterns suggesting venous outflow impairment than US^+^ results. Second, the agreement of V-PPG_*IR*_ with the forearm V-PPG_*R*_ was fair. Third and overall, fingertip V-PPG seems inadequate for detecting venous outflow impairment that is found at the forearm level, even in patients with positional venous compression at US imaging.

By the end of the 19th century, plethysmography was proposed in Physiology and Medicine for estimating volume changes in the limbs. Many techniques can be used to evaluate volume changes. Water immersion is one of the oldest tools but is inappropriate for upper limb recording during attitudinal tests. Strain gauge (Roos, 1969) plethysmography can be calibrated and expressed in volume increase normalized to the limb volume. Strain gauges are quite expensive, and the operators need to have a relatively large range of gauge lengths to adapt to individual anatomy. Air displacement (Coletta et al., 2001) or impedance (Nerurkar et al., 1990) plethysmography can also be proposed but remains more expensive than PPG. The use of red or infrared light combined with photosensitive detectors to estimate tissue light absorption of the finger was described in the late 1930s (Hertzman, 1937) and became popular in vascular physiology in the 1940s (Goldman and Schroeder, 1949). It was later computerized to enable routine use. In reflectance PPG, the emitting and receiving diodes are positioned close one to the other, allowing for the use of PPG on upper or lower limbs.

While there are many reports of arterial PPG from fingertip recording in TOS, at present, only one study in the 1980s proposed forearm V-PPG to estimate the presence or absence of venous outflow impairment (Antignani et al., 1990). The enormous development and diffusion of US imaging is probably the major reason why V-PPG was not adopted in clinical routine in the last decades. US imaging is a simple and widely available tool. Its advantage is that it can detect not only the presence but also the level of venous compression, as well as potential anatomical vascular variations (Leonhard et al., 2017). Nevertheless, some limits of US must not be neglected. A debate persists to define whether a positive US test is a sign of decreased velocity in the subclavian vein on abduction (Adam et al., 2018) or of the loss of atrial and respiratory dynamics (Longley et al., 1992). Furthermore, recent reports question the accuracy of venous ultrasonography whether in diagnosing upper limb thrombosis (Moore and Wei Lum, 2015; Brownie et al., 2020) or confirming venous compression without thrombosis in patients with suspected TOS (Butros et al., 2013; Povlsen and Povlsen, 2018). Lastly, venous US imaging does not correlate with forearm volume changes (Czihal et al., 2015). Therefore, US imaging cannot diagnose forearm edema on its own (Matsumura et al., 1997; Landry and Liem, 2007). In fact, collateral veins may normalize venous outflow despite complete positional occlusion of the subclavicular vein. In such cases, no upper limb swelling occurs as a possible cause of pain or discomfort. Finally, it is worth noting that when arterial compression was reported, some reports did not mention the presence or absence of venous compression at all and were then recorded as negative for venous positional compression.

It is clear that, compared with US, V-PPG provides strictly no information on the presence and localization of positional venous compression. This appears to be a major weakness of the V-PPG technique. Nevertheless and on the contrary, V-PPG recording confirming upper limb swelling is expected to better support the idea that positional subclavicular vein compression is responsible for the symptoms. For this specific reason, we do believe that the two techniques (i.e., US and V-PPG) add information one to the other. There are two possible other issues with V-PPG. First, PPG remains a semiquantitative technique. Although it correlates with strain gauge plethysmography (Louisy and Schroiff, 1995), it is not reliable in absolute values (van den Broek et al., 1989). Second, there have been multiple studies showing that R and IR PPG probes may show different responses to volume changes (Moco et al., 2018; Chatterjee and Kyriacou, 2019). These two points may also have appeared as important limitations to the use and diffusion of V-PPG. Specifically, with a semiquantitative tool, it was difficult to define the volume decrease to be expected from arm elevation, and whether the candlestick position resulted in the expected complete forearm emptying. The Ca + Pra maneuver changes the semiquantitative results of the Roos test (that includes a candlestick position only) to quantitative results. We believe that discordances between US and V-PPG should be interpreted neither as a lack of sensitivity of PPG because the presence of a venous compression at US does not necessarily induce forearm swelling, nor as a lack of specificity of PPG since we observed that some US reports just neglected the venous aspect when arterial imaging was positive. We put forward that V-PPG and US are complementary techniques. US detects venous compression but does not measure forearm swelling. V-PPG during the Ca + Pra maneuver is not a diagnostic test for subclavicular vein positional compression, but only (if a positional compression exists) for its hemodynamic consequences. While this could appear a limitation of the PPG technique, we believe that it is very interesting because it provides an objective argument for the presence of swelling during arm abduction as the cause of forearm discomfort in McCleery syndrome. In fact, when US of radio-vascular imaging shows the presence of venous attitudinal compression, it is unlikely that the venous compression itself results in symptoms if preclavicular collateral pathways normalize venous outflow.

The fair concordance between forearm V-PPG_*IR*_ and forearm V-PPG_*R*_ could result from the difference in light wavelengths; nevertheless, a fair to moderate relationship was predictable because a certain level of variability was expected from test–retest recordings. In fact, a difference by only a few degrees in abduction angles (or by a few centimeters of elbows in the sagittal plane) may result in venous compression rather than venous occlusion, or even in the absence of compression.

**FIGURE 4:**
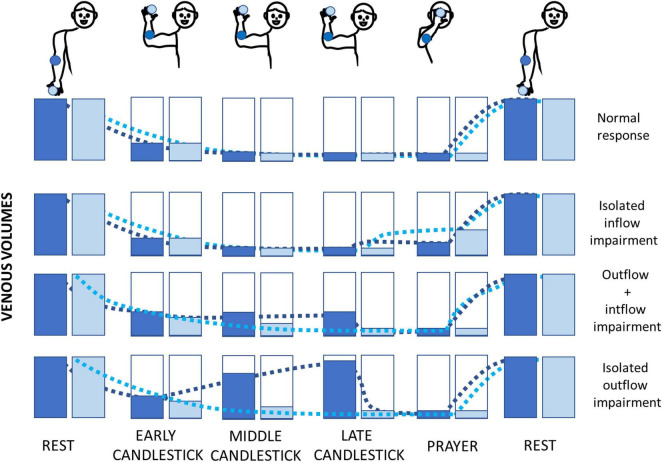
Schematic representation of the hypotheses of the fingertip (light blue) and forearm (dark blue) volume changes during the Ca + Pra maneuver. Normally, the forearm and fingertip volumes decrease with arm elevation, and refilling will only occur during arm lowering (upper figure). In case of ischemia (isolated inflow impairment), it is expected that the decrease in adrenergic tone due to postischemic vasodilation would be better observed distally (at the fingertip level). If simultaneous inflow and outflow impairments occur, emptying of the forearm is stopped while fingertip veins may drain into the forearm veins due to hydrostatic pressure. Emptying of the forearm will be completed during the prayer attitude. In cases of isolated venous outflow impairment, arterial inflow during the candlestick attitude will fill the forearm from bottom to top (as in a filling bottle) and will increase the volume at the forearm far before the fingertip volume increases. It is noted that fingertip volume would increase too if the duration of the candlestick attitude was long enough.

The question that remains is why did fingertip recordings fail to detect the presence of volume changes in any of the upper limbs where US found a venous compression, while many profiles suggesting outflow impairment were observed at the forearm level? We believe that it is logical as detailed below, and a schematic representation of our interpretation of the volume changes expected at the forearm and fingertip level during the Ca + Pra maneuver is presented in Figure 4.

• A normal arterial inflow and venous outflow (N) should result in almost similar V-PPG results at the forearm and fingertip level, with venous emptying in “Ca” and no significant change between “Ca” and “Pra.”
• If isolated arterial inflow impairment (A) occurs, fingertip and forearm volume would decrease to a minimum (veins should initially empty) during the candlestick phase. Then, during the “Pra” attitude, postischemic vasodilation at arterial decompression would be better observed distally (fingertip) than on the forearm.
• In case of simultaneous arterial inflow and venous outflow impairment (AV), finger veins would empty into the forearm veins, after which finger volume is minimal and forearm volume stabilizes after the initial emptying. Then, only forearm volume further decreases in the prayer phase. As a result, a fingertip probe will not detect AV impairment.
• If isolated venous outflow impairment (V) occurs without arterial inflow impairment (e.g., as a result of isolated occlusion of the subclavicular vein without collateral pathways), once again the fingertip vein drains into the forearm veins due to gravity, and fingertip volume reaches a minimum. Then, the volume increases from bottom to top in the elevated forearm (just as a progressively filling bottle), and volume changes are optimally detected at the forearm close to the elbow (Figure 4), while the finger veins should remain completely emptied (at least for a few seconds of tenth of seconds). Only forearm volume decreases to its minimum during the “Pra” position while fingertip volume is already minimal. Here again, a fingertip probe does not detect the isolated venous outflow impairment (except when the candlestick position is prolonged long enough to have the progressive upward filling of the forearm veins with persistent arterial inflow, reaching the fingers).

These are interpretations, some of which are pure assumptions (i.e., postischemic vasodilation to explain the A-type pattern), but some are logical as a consequence of gravity (i.e., filling from bottom to top, emptying of hand to forearm veins when arms are elevated). Although only hypotheses, they seem to explain our observations quite well. Finally, it could be suggested that in patients with Paget-Schroetter syndrome, venous emptying should be slowed when arms are rapidly elevated both at the fingertip and the forearm levels, with no significant change between “Ca” and “Pra.” This could not be observed in our experience because no patient with Paget Schroetter syndrome was included.

There are limitations to this study.

From a technical perspective, reported problems and sources of error with PPG are the individual variability in tissue to absorb, scatter, and reflect the emitted light (Blanc et al., 1993). These are not issues anymore during the Ca + Pra maneuver, with resting volume assumed to result from optimally filled veins (below heart level), and prayer position allowing for the optimal emptying of veins (above the heart level), defining the individual whole scale of minimal and maximal values. Simultaneous V-PPG_*IR*_ and V-PPG_*R*_ recordings might have been preferable to test the sole effect of wavelength difference on the results but were technically impossible, and interference between those two light-sensitive devices might have occurred despite differences in wavelengths.

From a methodological point of view, PPG can be contaminated by ambient light, cutaneous vasoconstriction and movements (Blanc et al., 1993). The former was limited by working in a moderately illuminated room. The latter is an issue mainly for the recording of small changes related to arterial pulsatility in arterial PPG recordings but shows the minor influence on V-PPG results, with PPG routinely used during muscle contraction at the low limb level (Tucker et al., 1998). It is important to note that the V-PPG test was performed sitting or standing while US imaging may have been conducted in the lying position. This might explain some underestimation of positive results with US since it was shown that positional compression of the subclavicular vessels is more frequent in the vertical than in the lying position (Cornelis et al., 2008). The relatively small number of subjects studied may limit the generalizability of our results, and future studies are required to confirm our observations and define whether differences may be observed between males and females, or during other provocative maneuvers. Finally, it could be suggested that clustering analysis applied to fingertip V-PPG recordings could result in specific fingertip V-PPG clusters and allow for the observation of four groups (as for the forearm) but with different mean curves. We believe that this is not the case because as previously explained and as shown in Figure 4, due to gravity emptying fingertip veins into forearm veins, our assumption is that the fingertip is just not the optimal location to detect outflow impairments.

From a physiological perspective, differences in anatomical vascular structure of the skin of the fingers compared with the skin of the forearm, as well as differences in innervation (in the fingers the vasomotor component is much higher than on proximal locations of the upper limb) may also result in the variations in volume being less visible at the fingertip compared to what can be seen on the forearm.

From a clinical perspective, it could be suggested that high central venous pressure may interfere with venous volume changes, as may be observed in cardiac failure or pulmonary hypertension. It cannot be excluded and requires further research. It could be also suggested that adrenergic vasoconstriction with positional pain will likely interfere with V-PPG results. If so, it is known that distal (i.e., hand or foot) vessels are more sensitive to adrenergic tone than vessels that are more proximal to the limbs. Therefore, recording at the forearm level is expected to be relatively insensitive to changes in adrenergic tone.

Finally, the limited number of subjects recruited in this pilot study limits the power of our results. Nevertheless, the total absence of profiles suggesting outflow impairment at the fingertip level, even in patients with US objective evidence of venous compression, was predictable (as explained in Figure 4), and we advocate that there is few if any need to further confirm this point in a larger population.

## Conclusion and Perspectives

Probe positioning on the forearm is essential if one aims to perform upper-limb V-PPG in patients with suspected TOS during the Ca + Pra maneuver. Both conceptually and as proven in this study, extracting the venous signal by a low-pass filter from fingertip PPG is inadequate for detecting volume changes suggesting outflow impairment. The additional value of forearm V-PPG in the diagnostic algorithm of patients with suspected TOS remains to be determined in a large population, but some immediate applications of the present findings may occur. Keeping in mind that the diagnosis of TOS remains difficult and its treatment is one of the most frequent causes of lawsuits in cardiothoracic surgery (Ferguson, 1982), we suggest that in addition to imaging, forearm V-PPG_*IR*_ during the Ca + Pra maneuver is easy and may provide recordable, objective arguments of forearm swelling in patients with suspected McCleery syndrome.

## Data Availability Statement

The raw data supporting the conclusions of this article will be made available by the authors, without undue reservation.

## Ethics Statement

The studies involving human participants were reviewed and approved by the CPP of Ile de France VII (protocol STOUT on 15 March 2018). The patients/participants provided their written informed consent to participate in this study.

## Author Contributions

JH, PR, MD, PA, and SH: recruitment. JH, PR, SD, MF, PA, and SH: data acquisition. JH, PR, SD, MF, MD, PA, and SH: data analysis. JH, PA, and SH: drafting the manuscript. PR, SD, MF, and MD: critical revision. All authors approved the final version of the manuscript.

## Conflict of Interest

The authors declare that the research was conducted in the absence of any commercial or financial relationships that could be construed as a potential conflict of interest.

## Publisher’s Note

All claims expressed in this article are solely those of the authors and do not necessarily represent those of their affiliated organizations, or those of the publisher, the editors and the reviewers. Any product that may be evaluated in this article, or claim that may be made by its manufacturer, is not guaranteed or endorsed by the publisher.

## Footnotes

1
https://www.graphpad.com
